# Development and Characterization of Simple Sequence Repeat Markers Providing Genome-Wide Coverage and High Resolution in Maize

**DOI:** 10.1093/dnares/dst026

**Published:** 2013-06-26

**Authors:** Jie Xu, Ling Liu, Yunbi Xu, Churun Chen, Tingzhao Rong, Farhan Ali, Shufeng Zhou, Fengkai Wu, Yaxi Liu, Jing Wang, Moju Cao, Yanli Lu

**Affiliations:** 1 Maize Research Institute, Sichuan Agricultural University, Wenjiang 611130, Sichuan, China; 2 Key Laboratory of Biology and Genetic Improvement of Maize in Southwest Region, Ministry of Agriculture, Wenjiang, China; 3 Institute of Crop Sciences, National Key Facilities for Crop Genetic Resources and Improvement, Chinese Academy of Agricultural Sciences, Beijing 100081, China; 4 International Maize and Wheat Improvement Center (CIMMYT), El Batan 56130, Texcoco, Mexico; 5 National Key Laboratory of Crop Genetic Improvement, Huazhong Agricultural University, Wuhan 430070, China; 6 Cereal Crops Research Institute (CCRI), Pirsabak 24100, Nowshera, Pakistan; 7 Triticeae Research Institute, Sichuan Agricultural University, Wenjiang 611130, Sichuan, China

**Keywords:** simple sequence repeat, whole-genome sequences, polymorphic SSR markers, teosinte, maize

## Abstract

Simple sequence repeats (SSRs) have been widely used in maize genetics and breeding, because they are co-dominant, easy to score, and highly abundant. In this study, we used whole-genome sequences from 16 maize inbreds and 1 wild relative to determine SSR abundance and to develop a set of high-density polymorphic SSR markers. A total of 264 658 SSRs were identified across the 17 genomes, with an average of 135 693 SSRs per genome. Marker density was one SSR every of 15.48 kb. (C/G)*n*, (AT)*n*, (CAG/CTG)*n*, and (AAAT/ATTT)*n* were the most frequent motifs for mono, di-, tri-, and tetra-nucleotide SSRs, respectively. SSRs were most abundant in intergenic region and least frequent in untranslated regions, as revealed by comparing SSR distributions of three representative resequenced genomes. Comparing SSR sequences and e-polymerase chain reaction analysis among the 17 tested genomes created a new database, including 111 887 SSRs, that could be develop as polymorphic markers *in silico*. Among these markers, 58.00, 26.09, 7.20, 3.00, 3.93, and 1.78% of them had mono, di-, tri-, tetra-, penta-, and hexa-nucleotide motifs, respectively. Polymorphic information content for 35 573 polymorphic SSRs out of 111 887 loci varied from 0.05 to 0.83, with an average of 0.31 in the 17 tested genomes. Experimental validation of polymorphic SSR markers showed that over 70% of the primer pairs could generate the target bands with length polymorphism, and these markers would be very powerful when they are used for genetic populations derived from various types of maize germplasms that were sampled for this study.

## 1. Introduction

Maize (*Zea mays* L.) is one of the most important food, feed, and industrial crops globally and a model system for the study of genetics, evolution, and domestication. The maize genome is large and complex. The estimated total size of genome draft is 2.3 Gb, with over 80% of repeated sequences of various types.^1^ The genetic variability in the maize genome can be utilized to enhance biotic and abiotic stress tolerance and to improve agronomic traits such as quality, maturity, and yield potential. Types of variation at the whole-genomic level include microsatellites or simple sequence repeats (SSRs), single-nucleotide polymorphisms (SNPs), insertions and deletions (indels), and various types of structure variation.

SSRs are tandemly repeated mono-, di-, tri-, tetra-, penta-, and hexa-nucleotide sequence motifs flanked by unique sequences.^2,3^ The unique sequences bordering the SSR motifs provide templates for specific primers to amplify SSR alleles via polymerase chain reaction (PCR), and allelic differences are usually the result of variable numbers of repeat units within a microsatellite structure.^4^ A larger number of repeated units is generally related to greater genotypic variation, and the shorter motifs such as those with mono-, and di-nucleotides usually possess more repeats than longer motifs such as those with tetra-, penta-, and hexa-nucleotides. However, shorter motifs can produce more slipped-strand mispairing (stuttering) during PCR, which usually lead to genotyping errors.^5,6^ Based on the repetitive architecture, purity, and complexity of their motifs, SSRs can be classified as perfect (single motif in an uninterrupted array), imperfect, or compound (two or more motifs in interrupted or uninterrupted arrays). As we known, SSR loci with longer or perfect motifs can exhibit a higher level of allelic variability.^5^

SSRs have been the genetic markers of choice, because they are easy to score, and have multiallelic nature, co-dominant inheritance, and clear advantages over restriction fragment length polymorphism and amplified fragment length polymorphism markers in terms of technical simplicity, throughput level and automation.^7^ Compared with SNP markers that are generally biallelic,^8^ SSR markers are more informative because it can detect multiple alleles per locus, so they are still commonly used nowadays.

Thanks to the availability of whole-genome or transcriptome sequences in public databases and in the recent advent of bioinformatics tools, development of genetic markers including SSRs has become much easier and more cost-effective. Genetic markers can be obtained by screening genomic, cDNA sequences, or libraries of clones. To facilitate access to and utilization of SSR markers in *Brachypodium*, 27 329 SSR markers were successfully designed through genome-wide analysis, but only 398 SSR markers have been developed from its bacterial artificial chromosomes end and expressed sequence tag databases.^9^ The availability of the completed soybean whole-genome sequence also provided an ideal resource for the genome-wide development of locus-specific SSR markers, and 33 065 high-polymorphic SSRs were developed with the availability of their genome positions and primer sequences.^10^ Barchi *et al.*^11^ combined the recently developed a restriction site-associated DNA approach with Illumina DNA sequencing to rapidly discover a large number of SNP and SSR markers for eggplant. Huang *et al*.^12^ identified over 3.6 million SNPs by sequencing 517 rice landraces, which were used in genome-wide association studies for 14 agronomic traits. These results show that genetic markers such as SSRs and SNPs are abundant in different crop genomes and can be easily scored, making it more accessible to the breeders and geneticists.

SSRs are abundant and well distributed throughout the maize genome, which can be employed as a preferred marker system. SSR markers have been utilized extensively in maize to characterize the genetic structure and diversity, to construct phylogenetic trees and to define potential heterotic groups, and to identify unique sources of allelic diversity.^13–15^ Furthermore, SSR markers have been widely used for genetic map construction, quantitative trait locus (QTL) mapping, map-based cloning, and marker-assisted selection (MAS) because of their ubiquity and high level of polymorphism. Hence, enriching the current maize linkage maps with more SSR markers is of great value for the global maize molecular breeding.

In recent years, many SSR markers have been developed and are publicly available (http://www.maizegdb.org/ssr.php) based on their target sequences among different maize germplasm accessions. However, a relatively low level of polymorphism was observed between cultivated maize and their relatives, and within populations derived from cultivated × teosinte and temperate × tropical maize crosses. The availability of the reference genome sequence and increasingly cost-effective sequencing facilities makes it possible to do whole-genome sequencing for more maize germplasm accessions. We used whole-genome sequence information from 3 typical tropical maize inbreds, 13 typical temperate maize inbreds from different heterotic groups, and 1 teosinte line, to analyse their genetic variation and to develop polymorphic molecular markers that can be used for high-resolution MAS, genomic selection, and QTL mapping. Using germplasm of diverse resources including teosinte and different types of maize lines, we can reveal and utilize unique alleles and loci better. Thus, the objectives of this study were to determine the abundance and characterization of SSRs in the maize genome and to use stringent screening to develop highly polymorphic SSR markers.

## 2. Materials and methods

### 2.1. Plant materials

Sequence data were generated for 17 genotypes including 16 improved maize inbred lines and 1 wild relative, *Z. mays* ssp. *mexicana* (hereafter Z. *mexicana*), which were listed in Table 1. Among the 16 improved maize inbreds, CML411 and P1 from International Maize and Wheat Improvement Center (CIMMYT) and 81565 from China were chosen to represent tropical/subtropical germplasm, ES40 was derived from traditional Chinese landrace, and the remaining 12 temperate maize inbreds were chosen to represent different heterotic groups in Chinese temperate maize. Temperate maize lines 178, Huangzao4, Ye478, Zheng22, and B73 representing PB, SPT, PA, LRC, and BSSS heterotic groups, respectively, were widely used for commercial hybrid production. For marker validation, additional six teosinte species obtained from United States Department of Agriculture (USDA) and four maize inbreds were also included (Table 1).

**Table 1.** Maize genotypes used in the study

Genotypes	Pedigree	Adaptation
178^a^	Selected from an introduced hybrid	Temperate
18Red^a^	American hybrid P78599	Temperate
18White^a^	American hybrid P78599	Temperate
48-2^a^	Synthesized population	Temperate
A318	Improved S37	Tropical
B73^a^	BSSS	Temperate
Dan598^a^	(Dan340 × Danhuang11) × (Danhuang02 × Dan599)	Temperate
ES40^a^	Landrace Linshuidadudu selected from Sichuan	Temperate
Han21^a^	American hybrid P78599	Temperate
Huangzao4^a^	Improved from Landrace, TangSiPingTou	Temperate
Lu9801	Ye502 × H21	Temperate
Mo17^a^	C103 × 187-2	Temperate
RP125^a^	Derived from hybrid Chuandan9	Temperate
Ye478^a^	U8112 × Shen5003	Temperate
Zheng22^a^	(Duqing × E28) × Lu Jiu Kuan	Temperate
81565^a^	(Huobai × Jin03)S2 × Heibai94	Tropical/subtropical
CML411^a^	P28C7-S4-#-BBBBBBBBBBB	Tropical/subtropical
CML206	[EV7992#/EVPO44SRBC3]#BF37SR-2-3SR-2-4-3-BB	Tropical/subtropical
CML85	P34C5F21-2-#1-2-2-#	Tropical/subtropical
P1^a^	Unknown	Tropical/subtropical
*Z. mexicana*^a^	*Zea mays* ssp. *mexicana*	Tropical
*Z. parviglumis*	*Zea mays ssp. parviglumis*	Tropical
*Z. huehuetenangensis*	*Zea mays ssp. huehuetenangensis*	Tropical
*Z. nicaraguensis*	*Zea nicaraguensis*	Tropical
*Z. luxurians*	*Zea luxurians*	Tropical
*Z. perennis*	*Zea perennis*	Tropical
*Z. diploperennis*	*Zea diploperennis*	Tropical

All the materials were used for experimental validation.

^a^Materials were only used for SSR identification and markers development. The chromosome number is 2*n* = 40 for *Z. perennis* and 2*n* = 20 for other species.

### 2.2. Maize genome sequences

For maize lines 81565, 18Red, 18White, 48-2, Dan598, ES40, RP125, and Z. *mexicana*, sequences were generated, and paired-end libraries were constructed according to the Illumina manufacturer's instructions. An average resequencing depth was 13× and genome coverage was 85% for maize inbreds. One teosinte species *Mexican* was sequenced with an average of resequencing depth of 9× and genome coverage of 74%. The genome sequences for the remaining maize lines were downloaded from the NCBI Sequence Read Archive (SRA) database (SRA049859 and SRA051245) and NCBI GenBank (JQ886798–JQ887980). All sequence reads were aligned against the maize B73 reference genome (www.maizesequence.org Release 4a.53) using Short Oligonucleotide Alignment Program 2 (http://soap.genomics.org.cn/). Sequencing and reads mapping were carried out at Beijing Genomics Institute (Shenzhen, China).^16–18^

### 2.3. SSR identification and primer design

SSR motifs were identified in 17 genomes using MISA (MIcroSAtellite identification tool) program downloaded from the Leibniz Institute of Plant Genetics and Crop Plant Research website (http://pgrc.ipk-gatersleben.de/misa/). Only perfect SSRs including mono-, di-, tri-, tetra-, penta-, and hexa-nucleotide motifs with numbers of uninterrupted repeat units more than 10, 7, 6, 5, 4, and 4, respectively, were targeted. The 5′- and 3′-untranslated regions (UTR), protein coding sequence (CDS), intron, and intergenic regions were determined based on their original annotations of the maize B73 reference genome (www.maizesequence.org Release 4a.53). Promoter sequences were determined at 2 kb upstream of the transcription initiation site.

Any SSR locus to be used to develop genetic markers should include a perfect repeat motif and two unique flanking sequences with 300 bp on each sides of the repeat. In our study, SSR candidate sequences were used for BLASTN search against the genome sequences (*e*-value cut-off of 1*e*^−10^), and filtered with >90% of identity and minimum alignment length with >85% of the flanking sequences. Those with unique hit, together with their specific flanking sequences, were identified as candidate SSR loci. Then, we wrote a Perl script to combine SSRs within 5 kb of different genomes with the same motif and to identify polymorphic SSR loci among 17 genotypes depending on the presence of motifs.

The forward and reverse primers were designed based on unique flanking sequences using Primer 3 (http://primer3.sourceforge.net/). Input parameters for the primer design were as follows: minimum, maximum, and optimal sizes were 18, 27, and 20 nt; minimum and maximum GC were 20 and 80%; and minimum, maximum, and optimal *T*_m_ were 57, 63, and 60°C, respectively. The deviation of amplicon size of each SSR primer ranged from 30 to 500 bp based on the expected SSR sequence length.

In addition, electronic polymerase chain reaction (e-PCR) programme (http://www.ncbi.nlm.nih.gov/projects/e-pcr/) was applied to check the uniqueness and specificity of designed primers in the genomes. The parameters were set as following: the word size was 9, the discontiguous word was 1, the maximal allowed deviation of hit product size was 100, the maximum mismatches allowed, and the maximum indels allowed were 1, respectively. On the other hand, the published SSR markers reposited in MaizeGDB (http://www.maizegdb.org/) were downloaded and amplified *in silicon* through e-PCR programme for further comparison.

### 2.4. Experimental validation of polymorphic SSR markers

To assess the value of identified SSR markers, 151 primer pairs from 10 chromosomes including all types of SSR were chosen for experimental validation. The samples used in this experiment included 20 improved maize lines and 7 teosinte lines (Table 1). Genomic DNA was extracted from seedlings using the CTAB method. Primers were made by Shanghai DNA Biotechnologies Co., Ltd. PCR was performed in 25 μl reactions containing 2.5 μl buffer, 2.5 μl MgCl_2_ (25 mM), 4.0 μl dNTP (2.5 mM), 0.2 μl Taq polymerase (5 U/μl), 1 μl template DNA (100 ng/μl),13.8 μl ddH_2_O, and 0.1 μg primers. The PCR conditions were as follows: 1 cycle at 94°C for 5 min; 35 cycles at 94°C for 30 s, 60°C for 30 s, 72°C for 1 min, and 1 cycle at 72°C for 10 min. PCR products mixed with loading buffer were heated at 95°C for 5 min and quickly chilled on ice. The entire mixture was electrophoresed on 6% denaturing polyacrylamide gel, and the genotype was scored after silver staining. The number of alleles was recorded and the polymorphism information content (PIC) was calculated as described by Smith *et al.*^19^

## 3. Results

### 3.1. The abundance of SSRs in the maize genome

A large number of perfect SSRs with mono-, di-, tri-, tetra-, penta-, and hexa-nucleotide motifs were identified, but the numbers varied among different genomes (Table 2). The average number of SSRs was 135 693 in 17 genotypes, ranging from 133 346 loci observed in *mexicana* to 136 723 loci in tropical/subtropical maize inbred 81565. Some reads from Z. *mexicana* could not be mapped onto the reference genome, which resulted in relatively lower genome coverage and thus, less SSRs identified compared with other maize inbreds. A total of 264 658 unique SSR loci were detected in 17 genomes, of which mono-, di-, tri-, tetra-, penta-, and hexa-nucleotide SSRs were 153 231, 65 236, 25 910, 6572, 8839, and 4870, respectively. The mono-nucleotide motif is the most abundant, accounted for 57.90%. There were 38 971 common SSRs (15% of the total) observed to be the same across 17 genotypes. The SSR density was calculated based on the maize reference genome size of 2.1 Gb, and there was a little difference among 17 genotypes for each nucleotide motif, with an average interval of 15.48 kb between SSR loci for every genome. However, the average intervals for mono-, di-, tri-, tetra-, penta-, and hexa-nucleotide SSRs were remarkably different, which were 26.93, 60.88, 150.95, 717.70, 505.05, and 942.55 kb, respectively (Table 2). SSRs were considerably abundant and distributed throughout the maize genome, with a small average marker interval (7.93 kb) for all detected loci.

**Table 2.** Numbers and density of SSR loci identified in 17 maize genomes

Genotypes	SSR numbers							SSR interval (kb)						
	MNR	DNR	TNR	TTR	PNR	HNR	Total	MNR	DNR	TNR	TTR	PNR	HNR	Total
178	78 367	34 604	13 929	2 964	4172	2226	136 262	26.80	60.69	150.76	708.50	503.36	943.40	15.41
81565	79 042	34 457	13 893	2929	4178	2224	136 723	26.57	60.95	151.16	716.97	502.63	944.24	15.36
18White	77 144	34 451	13 931	2928	4166	2217	134 837	27.22	60.96	150.74	717.21	504.08	947.23	15.57
18Red	77 049	34 495	13 919	2933	4188	2221	134 805	27.26	60.88	150.87	715.99	501.43	945.52	15.58
48-2	76 920	34 448	13 884	2924	4123	2219	134 518	27.30	60.96	151.25	718.19	509.34	946.37	15.61
B73	77 888	34 755	14 028	2948	4181	2239	136 039	26.96	60.42	149.70	712.35	502.27	937.92	15.44
CML411	78 591	34 546	13 900	2935	4189	2225	136 386	26.72	60.79	151.08	715.50	501.31	943.82	15.40
Dan598	78 558	34 559	13 954	2904	4159	2227	136 361	26.73	60.77	150.49	723.14	504.93	942.97	15.40
ES40	78 978	34 372	13 894	2924	4161	2221	136 550	26.59	61.10	151.14	718.19	504.69	945.52	15.38
Han21	78 539	34 584	13 951	2898	4157	2233	136 362	26.74	60.72	150.53	724.64	505.17	940.44	15.40
Huangzao4	76 773	34 445	13 878	2907	4165	2224	134 392	27.35	60.97	151.32	722.39	504.20	944.24	15.63
Mo17	78 360	34 442	13 910	2902	4108	2220	135 942	26.80	60.97	150.97	723.64	511.20	945.95	15.45
P1	78 975	34 447	13 849	2961	4146	2235	136 613	26.59	60.96	151.64	709.22	506.51	939.60	15.37
RP125	76 880	34 523	13 934	2929	4198	2244	134 708	27.32	60.83	150.71	716.97	500.24	935.83	15.59
*Z. mexicana*	75 997	34 339	13 806	2886	4085	2233	133 346	27.63	61.15	152.11	727.65	514.08	940.44	15.75
Ye478	78 571	34 508	13 973	2944	4159	2234	136 389	26.73	60.86	150.29	713.32	504.93	940.02	15.40
Zheng22	78 921	34 455	13 870	2920	4156	2230	136 552	26.61	60.95	151.41	719.18	505.29	941.70	15.38
Average	77 974	34 496	13 912	2926	4158	2228	135 693	26.93	60.88	150.95	717.70	505.05	942.55	15.48
Total	153 231	65 236	25 910	6572	8839	4870	264 658	13.70	32.19	81.05	319.54	237.58	431.21	7.93
Common	22 453	9963	4603	553	929	470	38 971	93.53	210.78	456.22	3797.47	2260.50	4468.09	53.89

MNR, DNR, TNR, TTR, PNR, and HNR indicate mono-, di-, tri-, tetra-, penta-, and hexa-nucleotide SSRs.

We also examined different SSR repeat types in the genome for all tested genotypes. The frequencies of different nucleotide repeat types in each motif were different, but they showed similar frequency patterns in different genomes. Here, we compared the frequencies of different SSR repeat types by taking the reference line B73 as an example (Table 3). Of mononucleotide motifs, C/G repeats accounted for ∼54.4%, which was slightly higher than A/T repeats. Of the di-nucleotide motifs, (AT)*n* were most frequent (24.93%), followed by (AG/CT)*n* (24.36%), (TA)*n* (21.03%), and (GA/TC) (20.64%), while the (CG)*n* motif was least frequent (0.80%). Of the tri-nucleotide motifs, (CAG/CTG)*n* was the most abundant (15.86%), while other nucleotide repeat types had lower frequencies (0.4–6%). Of the tetra-nucleotide SSRs, (AAAT/ATTT)*n*, was most frequent (10.35%), and the frequencies for the rest nucleotide repeat types were all lower than 7%. There were many types of penta- and hexa-nucleotide SSRs, each with low frequencies, ranging from 0.04 to 4%. The numbers of mono-, di-, tri-, tetra-, penta-, and hexa-nucleotide motifs in different repeat unit classes are also listed in Table 3. The average repeat lengths were different among various motifs ranging from 11.02 for (A/T)*n* to 58.84 for (AGT/ACT)*n*.

**Table 3.** Number of SSRs in different repeat classes in the maize genome B73

Motifs	Repeats number									Total	Average repeat number	Average repeat length (bp)
	<5	5–7	8–10	11–15	16–20	21–25	26–30	31–40	>40			
G/C	0	0	13 085	24 297	3 832	866	243	45	1	42 369	12.31	12.31
A/T	0	0	22 410	11 687	1114	192	57	32	27	35 519	11.02	11.02
AT	0	1875	3268	1379	708	478	378	466	111	8663	13.28	26.56
CT/AG	0	3615	3504	740	265	127	72	95	47	8465	9.42	18.84
TA	0	1725	2417	1087	625	465	364	492	134	7309	14.03	28.05
GA/TC	0	3220	2938	519	237	103	71	57	28	7173	9.19	18.39
CA/TG	0	666	542	72	15	5	1	5	1	1307	8.24	16.48
GT/AC	0	582	588	73	12	5	4	4	0	1268	8.32	16.64
GC	0	223	66	3	3	0	0	0	0	295	7.42	14.83
CG	0	219	55	0	1	0	0	0	0	275	7.29	14.59
CAG/CTG	0	1980	242	3	0	0	0	0	0	2225	6.5	19.51
GAT/ATC	0	526	264	46	5	1	1	0	0	843	7.49	22.48
GCA/TGC	0	711	99	0	0	0	0	0	0	810	6.42	19.25
GAC/GTC	0	587	184	28	1	0	0	0	0	800	7.02	21.06
ATT/AAT	0	352	124	65	42	28	18	10	4	643	10.25	30.74
TTA/TAA	0	359	96	66	32	27	24	14	2	620	10.31	30.94
CGT/ACG	0	429	131	34	0	0	0	0	0	594	7.18	21.55
TGA/TCA	0	350	167	58	9	3	1	0	0	588	7.86	23.59
CGA/TCG	0	474	100	4	0	0	0	0	0	578	6.69	20.06
CGC/GCG	0	505	49	4	1	0	0	0	0	559	6.5	19.51
GCC/GGC	0	490	42	5	0	0	0	0	0	537	6.49	19.46
TAT/ATA	0	246	103	43	47	35	13	13	5	505	11.39	34.16
CGG/CCG	0	416	48	3	0	0	0	0	0	467	6.5	19.51
TAC/GTA	0	232	50	26	16	6	1	5	1	337	8.54	25.63
TTG/CAA	0	230	80	16	4	0	0	0	0	330	7.32	21.96
TTC/GAA	0	288	26	9	0	0	1	2	3	329	7.42	22.26
ATG/CAT	0	260	45	11	4	0	1	0	0	321	7.01	21.04
GCT/AGC	0	291	16	1	1	0	0	0	0	309	6.36	19.09
TGG/CCA	0	280	24	2	0	0	0	0	0	306	6.4	19.2
TAG/CTA	0	186	49	26	11	10	8	3	3	296	9.63	28.9
CTC/GAG	0	232	46	6	0	0	0	0	0	284	6.82	20.45
CTT/AAG	0	245	31	1	1	1	0	1	1	281	7	21
TCC/GGA	0	193	43	8	1	0	0	0	0	245	6.91	20.74
CCT/AGG	0	187	32	5	2	1	0	0	0	227	6.93	20.79
CAC/GTG	0	198	18	0	1	0	0	0	0	217	6.46	19.38
ACC/GGT	0	191	16	1	0	0	0	0	0	208	6.39	19.17
ACA/TGT	0	125	58	10	0	0	1	0	0	194	7.34	22.02
AGA/TCT	0	169	21	2	0	1	0	0	0	193	6.58	19.74
GTT/AAC	0	85	29	4	0	1	1	0	0	120	7.45	22.35
AAAT/ATTT	0	288	15	2	0	0	0	0	0	305	5.58	22.33
AGGC/GCCT	0	153	11	4	0	0	0	0	0	168	5.9	23.6
TATT/AATA	0	195	10	2	0	0	0	0	0	207	5.72	22.88
TCGT/ACGA	0	126	0	0	0	0	0	0	0	126	5.07	20.29
TTAT/ATAA	0	111	16	0	0	0	0	0	0	127	5.88	23.53
TTTA/TAAA	0	157	7	2	0	0	0	0	0	166	5.67	22.67
CGAGC/GCTCG	153	24	0	0	0	0	0	0	0	177	4.16	24.95
TTTTA/TAAAA	82	28	0	0	0	0	0	0	0	110	4.29	25.75
ATTTT/AAAAT	69	35	1	0	0	0	0	0	0	105	4.42	22.1

SSR motifs with repeats number >100 in total were listed here.

### 3.2. Screening of SSR loci and development of maize SSR markers

A total of 2034 SSR markers have been recently developed and reported on MaizeGDB website (www.maizegdb.org). Among the public markers, 1556 SSRs have genomic positions. Through e-PCR programme conducted in B73 genome, 827 SSR markers have specific amplicon, 60 SSR markers have more than one binding sites, and the remaining markers have no proper binding sites on the 10 chromosomes. Here, we developed a new database containing more SSR markers with unique flanking sequences. From the SSRs that could be detected (264 658) across 17 maize genomes, 189 087 (71.45%) of them were identified with unique flanking sequences with an average of 82 741.9 SSR loci for each genome (Table 4). The average numbers of SSR loci with different motifs for each genome were notably different, accounting for 55.19, 74.60, 48.09, 81.94, 80.69, and 68.94% of the total SSRs for mono-, di-, tri-, tetra-, penta-, and hexa-nucleotide motifs, respectively (Table 4). It implies that over 80% of tetra- and penta- nucleotide motifs in the maize genome can be used to design SSR markers. A total of 25 437 SSRs with unique flanking sequences were found to be shared across 17 tested genomes, of which 9240 (36.33%) were polymorphic.

**Table 4.** Summary of SSR loci with unique flanking sequences identified in tested maize genomes

Motifs	Average SSRs		Total SSRs		Common SSRs		Common SSRs with polymorphism	
	No.	%^a^	No.	%^b^	No.	%^c^	No.	%
MNR	43 030.4	55.19	103 486	67.54	12 029	54.31	4588	38.14
DNR	25 733.2	74.6	52 876	81.05	8630	79.87	3776	43.75
TNR	6689.9	48.09	15 946	61.54	2755	59.53	560	20.33
TTR	2397.2	81.94	5658	86.09	652	83.27	142	21.78
PNR	3355.4	80.69	7481	84.64	950	83.11	129	13.58
HNR	1535.8	68.94	3640	74.74	422	74.17	45	10.66
Total	82 741.9	60.98	189 087	71.45	25 437	63.47	9240	36.33

MNR, DNR, TNR, TTR, PNR, and HNR indicate mono-, di-, tri-, tetra-, penta-, and hexa-nucleotide SSRs.

^a^Percentage of the average number of SSRs with unique flanking sequences against all for every tested maize genome.

^b^Percentage of total SSR number with unique flanking sequences against all identified in 17 maize lines.

^c^Percentage of the common loci against all that are the same in 17 maize lines.

**Figure 1.**
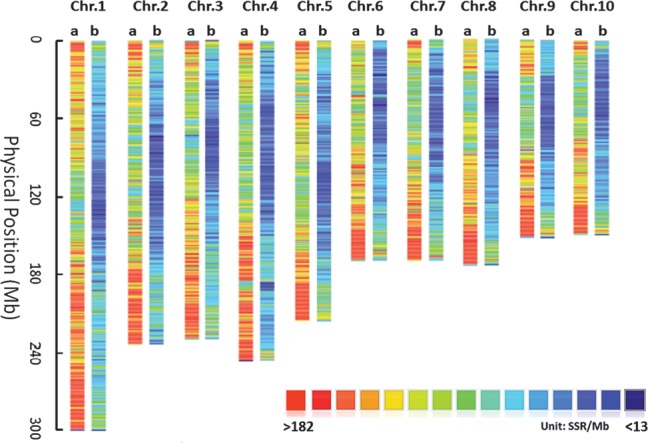
Distributions of 263,423 SSR loci (a) and 111,887 new developed SSR markers (b) with unique physical positions across 10 chromosomes in the B73 reference genome (www.maizesequence.org Release 4a.53). Different colors represent levels of density of SSRs.

Of 189 087 candidate SSRs, 188 571 loci have specific physical position and would be developed as genetic markers in the study. Primer pairs were then designed for the 188 571 SSR loci, with 13 344 (chromosome 10) to 29 779 (chromosome 1) SSRs on each chromosome, and 173 587 of them were polymorphic with length differences and present-absent variation in 17 genomes. E-PCR programme was further conducted to validate and refine the specificity of new designed SSR markers, and 111 887 primer pairs of them could bind as expected and the others were amplified with multiple binding sites or false match. Through comparing SSR sequences among 17 tested genomes, a new database was developed to include 111 887 SSR markers with specific physical positions, with proportion of 59% of the candidate SSR loci with specific flanking sequences (Table 5 and Supplementary Table S1). Among these markers, SSRs with mono-, di-, tri-, tetra-, penta-, and hexa- nucleotide motifs accounted for 58.00, 26.09, 7.20, 3.00, 3.93, and 1.78%, respectively. A total of 35 573 SSR loci, accounting for 31.8% of the refined SSR markers, showed length polymorphism in the 17 tested genotypes. The PIC for these polymorphic SSRs varied from 0.05 to 0.83, with an average of 0.31 (Supplementary Table S1). SSR markers with mono- and di-nucleotide motifs showed higher levels of polymorphism (33.87 and 37.31%, respectively) than other SSR markers with tetra-, penta-, and hexa-nucleotide motifs (7.44–17.19%). Comparing with the SSR markers in MaizeGDB database, there were 18 606 SSR markers, accounting for 16.6% of the newly developed SSR markers, shared the same loci with the public SSR markers with various motifs. However, only 527 (0.47%) newly developed SSR markers had completely compatible position with public SSR primers. Additionally, the average SSR lengths and number of loci across 10 chromosomes were calculated for three SSR datasets, all SSRs, SSRs with unique flanking sequences, and polymorphic SSRs (Fig. 1). In each of the three SSR datasets, the numbers of loci gradually declined with the increase of SSR lengths, the same as shown in previous studies.^20^

**Table 5.** Numbers of candidate SSR markers, and polymorphic SSR markers detected in 17 maize lines and previously developed SSR in MaizeGDB database

Chr	Candidate SSR markers												Poly. (%)^a^	SSRs in MaizeGDB database
	MNR		DNR		TNR		TTR		PNR		HNR			
	Mono-	Poly-	Mono-	Poly-	Mono-	Poly-	Mono-	Poly-	Mono-	Poly-	Mono-	Poly-		
1	6957	3422	2958	1737	1129	242	439	84	643	73	304	12	30.94	293
2	4875	2488	2118	1212	697	157	294	76	418	46	203	17	31.71	226
3	5079	2414	2289	1217	777	177	330	69	514	49	209	17	30.01	224
4	4831	2331	2246	1264	774	166	331	73	422	45	192	23	30.73	141
5	4623	2498	1851	1140	681	164	276	67	461	55	198	18	32.76	146
6	3501	1816	1499	844	579	118	248	44	345	25	158	12	31.11	111
7	3548	1870	1473	929	505	150	271	41	285	44	150	12	32.83	112
8	3553	1845	1391	901	504	118	185	32	315	50	180	13	32.56	118
9	3059	1726	1262	847	451	100	203	39	297	40	137	16	33.85	102
10	2884	1572	1214	803	436	127	203	52	248	26	111	8	33.68	83
Total	42 910	21 982	18 301	10 894	6533	1519	2780	577	3948	453	1842	148	31.79	1556

MNR, DNR, TNR, TTR, PNR, and HNR indicate mono-, di-, tri-, tetra-, penta-, and hexa-nucleotide SSRs.

Mono: monomorphism; poly: polymorphism; Chr: chromosome.

^a^Percent of polymorphic SSR markers over all of candidate SSR markers *in silicon* analysis.

### 3.3. Distribution of SSRs in different genomic regions

**Figure 2.**
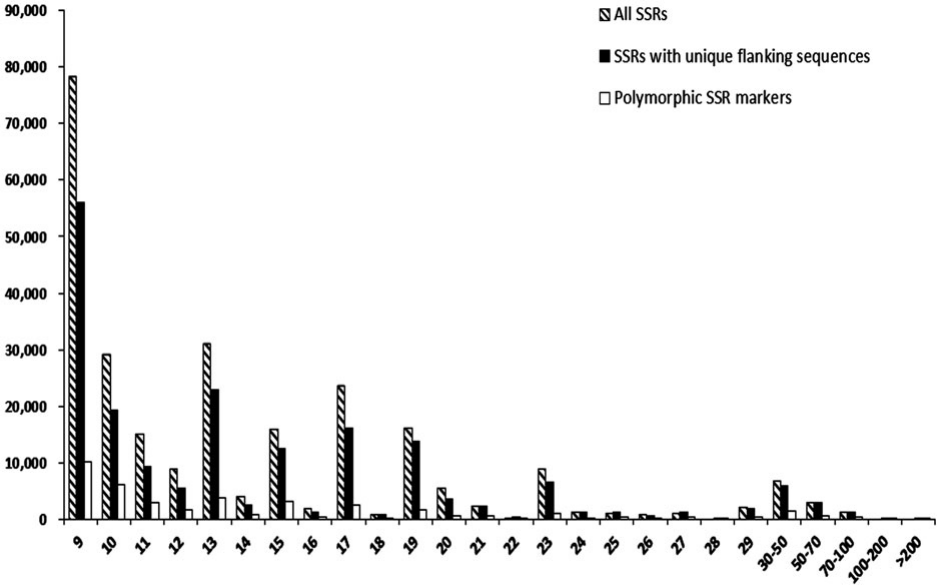
Correlation between SSR numbers and SSR lengths.

A total of 264 658 SSRs were detected in 17 genomes, and 263 423 loci of them have specific physical position. The distributions of 263 423 SSR loci and 111 887 newly developed SSR markers refined by e-PCR programme across 17 tested genomes were shown in Fig. 2 a and b , respectively. SSRs were unevenly distributed on chromosome regions, and there were much more loci located in near telomeric regions than near centromeres, which was accordance with the distribution patterns of genes in maize.^21^ Moreover, we compared SSR distributions across five genomic regions using tested genomes of P1, B73, and *Z. mexicana* to represent tropical, temperate, and wild maize germplasm, respectively (Table 6). SSR loci were most abundant in intergenic region and least frequent in UTR region. Polymorphism rate and GC content of SSRs in coding regions were higher than other genic regions.

**Table 6.** The distribution of SSRs in different genomic regions

	All SSR loci				SSR loci with unique flanking sequences				SSR loci with polymorphism			
	Count	Interval (kb)	Length (bp)	GC%	Count	Interval (kb)	Length (bp)	GC%	Count	Interval (kb)	Length (bp)	GC%
B73												
5′-UTR	3804	6.91	16.10	32.20	3601	7.30	16.19	32.60	1335	19.69	16.23	26.55
3′-UTR	3829	6.87	16.13	33.68	3628	7.25	16.22	33.94	1341	19.62	15.90	26.70
CDS	2553	17.27	19.80	74.82	2391	23.10	19.87	75.28	309	178.73	21.28	56.84
Intron	11 857	10.13	16.03	29.94	11 038	10.88	16.12	29.97	4704	25.54	16.70	29.60
Promotors	10 370	6.26	17.35	27.57	9248	7.02	17.54	26.71	3344	19.42	17.26	20.82
Intergenic	107 658	21.63	16.17	50.49	65 098	28.56	17.12	46.22	21 650	85.88	17.70	42.24
Total/average	136 039	15.15	16.33	47.01	90 441	22.79	17.11	42.62	30 900	66.70	17.49	37.61
*Z. Mexicana*												
5′-UTR	2520	10.25	15.55	26.23	2263	11.42	15.66	24.10	1007	25.66	14.35	17.18
3′-UTR	3329	7.76	17.34	38.34	3092	8.35	17.42	38.10	1100	23.48	15.81	23.81
CDS	3636	14.29	17.70	45.01	3252	15.98	17.93	43.96	954	54.47	16.14	23.08
Intron	8102	14.23	16.24	32.41	7168	16.08	16.48	30.43	3035	37.98	15.63	24.71
Promotors	10 903	5.86	17.79	34.35	10 035	6.37	17.95	34.04	3627	17.62	16.32	21.71
Intergenic	107 785	16.50	16.18	49.95	57 813	30.76	17.51	42.07	22 413	79.33	16.64	38.84
Total/average	133 346	15.46	16.38	47.13	80 545	25.59	17.50	39.82	30 787	66.94	16.45	34.45
P1												
5′-UTR	2684	9.60	15.27	24.75	2419	10.65	15.37	22.50	926	27.82	15.44	20.02
3’-UTR	3478	7.41	17.04	37.07	3230	7.98	17.11	36.90	1044	24.68	16.96	29.53
CDS	3724	13.91	17.44	44.49	3351	15.46	17.63	43.48	911	56.86	17.51	28.36
Intron	8622	13.32	15.96	31.18	7659	14.99	16.14	29.13	3073	37.36	16.28	28.44
Promotors	11 218	5.68	17.56	33.82	10 316	6.18	17.67	33.50	3486	18.28	17.56	24.74
Intergenic	110 043	16.17	16.06	49.51	59 167	30.07	17.26	41.81	22 475	79.16	17.39	41.03
Total/average	136 613	15.09	16.24	46.58	82 831	24.88	17.24	39.38	30 621	67.31	17.28	37.09

The average intervals between SSRs were the longest in intergenic regions, second in CDS regions, and smallest in promotors (Table 6). Distributions for the SSRs with unique flanking sequences and for the polymorphic SSRs across tested genomes also varied among the six genomic regions, but the trend was consistent with that for all the candidate SSR loci. This result is also in full agreement with a previous report in rice.^22^ In addition, SSR distribution was rather similar among the three representative genotypes.

Furthermore, the repeat types of SSRs in CDS region of B73 were investigated. SSRs with tri-nucleotide repeats were the most (1832) among the six repeat types, with proportion of 71.8% in CDS region. The tri- and hexa-nucleotide SSRs that would not bring the frame shift accounted for 84.1% (2148) of the SSRs in CDS region. Therefore, only 15.9% of the SSRs in CDS region have potential threats to the gene structure.

### 3.4. SSR markers validated for quality and polymorphism

**Figure 3.**
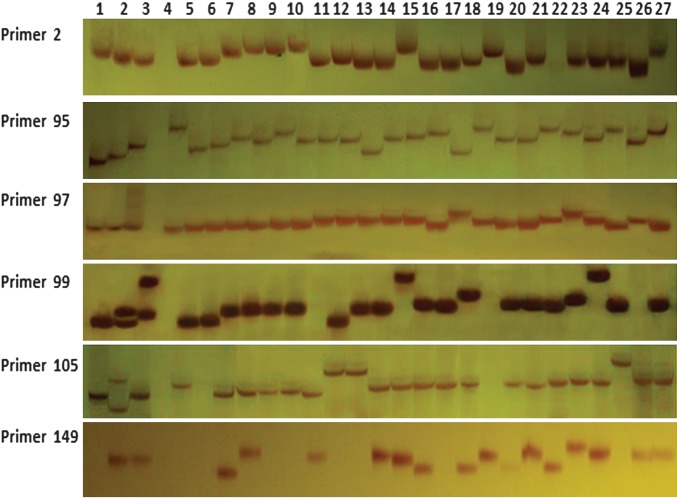
Experimental validation of six randomly selected SSR markers in 27 genotypes. Lanes 1–27 were PCR products of *Zea perennis*, *Z. diploperennis*, *Z. mays* ssp. *parviglumis*, *Z. mays* ssp. *huehuetenangensis*, *Z. nicaraguensis*, *Z. luxurians*, *Z. mays* ssp. *mexicana*, RP125, 18Red, 18White, CML206, 81565, A318, P1, Han21, CML85, CML411, Ye478, Mo17, Zheng22, 178, 48-2, B73, Lu9801, ES40, Huangzao4, and Dan598, respectively.

A total of 151 SSR markers were randomly chosen for experimental validation using 20 maize inbreds and 7 teosinte lines (Fig. 3 and Table 1). Of them, 121 primer pairs (80.1%) generated specific products and distinct bands, while 30 primer pairs failed to produce stable or clear bands due to the lack of sequence specificity in the genomic DNA samples. The majority of the 121 primer pairs (112 primer pairs) revealed high levels of allelic diversity in tested 27 lines, with PIC values of 0.074–0.796 (an average of 0.478). The 112 polymorphic SSR loci contained 329 alleles in total and an average of 2.94 alleles with a range of 2–5 (Supplementary Table S2).

In addition, we made a detailed comparison of the allele number and PIC value *in silicon* analysis and in maker validation in 17 tested genotypes. Forty-one of the 121 primers possessed the practical alleles in accordance with the expected alleles, 38 primer pairs had more allele number, and 42 primer pairs had less allele number *in silicon* analysis than in maker validation (Supplementary Table S2). Additionally, comparing polymorphism *in silicon* analysis using 17 tested genomes and in maker validation using 27 genotypes, we found that 51 primer pairs showed more alleles and higher PIC values in validation experiment (Supplementary Table S2). Interestingly, 26 of 151 SSR markers with no polymorphism *in silicon* analysis showed more than one alleles in validation experiment. We also found that the length of PCR products *in silicon* analysis almost consist with those in marker validation (Supplementary Tables S1 and S2). The results indicate that newly developed SSR markers are informative and useful, and 70% of the SSR markers in our database are valid and polymorphic.

## 4. Discussion

SSRs are co-dominant, abundant, high polymorphic, and dispersed throughout plant genomes. Based on the survey across genomes, on average one SSR was found every 1.14 kb in *Arabidopsis*,^23^ 3.6 kb in rice,^22^ 4 kb in *Brassica oleracea*,^24^ 4.5 kb in soybean,^10^ 220 kb in sorghum,^25^ and 578 kb in wheat.^26^ In this study, average SSR density was one SSR every 15.48 kb. These may reflect real genetic differences existing among plant genomes at DNA level, and also the differences involved in sequencing methods and procedures. We used maize inbred B73 as the reference genome, some reads from maize wild relative, Z. *mexicana*, could not be mapped onto the reference, resulting in a relatively lower genome coverage and thus, less SSRs identified compared with other maize inbreds. Therefore, the number of SSR loci identified from Z. *Mexicana* may be underestimated.

In general, a small difference in SSR distribution was found for different populations or ecotypes in the same species. For instance, a very similar SSR distribution was found between indica and japonica rice, and the SSR density (interval between two SSRs) varied from one SSR every 2.0–8.1 kb, which was higher in 5′-UTR (one SSR every 2.1 and 2.0 kb, respectively) but low in CDS regions (one SSR every 8.1 and 7.7 kb, respectively).^22^ Our study revealed a similar SSR distribution pattern across the tested temperate, tropical, and wild maize lines. However, SSRs are not evenly distributed in different genomic regions with much lower SSR density in CDS region than in UTR and intronic regions. Intriguingly, we found that majority of SSRs resided in CDS region were tri-nucleotide repeats, which was consistent with other report and implied the specific selection against frame shift mutations in coding regions.^27^ Comparing with rice genome, the average SSR length was approximately identical (16–17 bp), but the average GC content in maize SSR sequences was much higher (47%) than rice (27%). In the maize genome, the proportions of mono-, di-, and tri-nucleotide SSR motifs were ∼60, 20, and 10%, respectively. Tetra-, penta-, and hexa-nucleotide SSR motifs were less abundant, together accounting for 10%, which was accordance with the report in rice.^22^ SSR densities for different motifs were also unbalanced and the average interval varied from 26 to 950 kb. Moreover, we found that C/G, AT, and CAG/CTG repeats were the most common for mono-, di-, and tri-nucleotide SSRs, respectively, in maize, while A/T, AG, and AGG/CCT repeats are the most common in rice.^22^ Meanwhile, AT repeats were also the most common dinucleotide motifs in sorghum.^25^

Short-read data from next-generation sequencing technologies are now being generated across a range of research projects. The fidelity of this data can be affected by several factors, and mapping errors and gaps still exist to a certain extent.^28^ However, the availability of the maize genome sequence still affords us a simple and economical way to survey and identify markers, thus enabling us to develop more convenient molecular markers for breeding applications. Several sets of maize germplasm including temperate, tropical, and their wild relatives were resequenced using next-generation sequencing technology.^18,29–31^ There are two major advantages in using currently available data for the analysis of SSR distribution and marker development. The maize germplasms from different ecological regions and heterotic groups (PB, SPT, PA, LRC, and BSSS) are highly diverse and host rare and unique alleles, providing an opportunity of using these types of genetic variation in hybrid maize breeding. On the other hand, whole-genome sequence data provide an ideal resource and the most complete picture of genetic variation for developing high-density genetic markers.

SSR markers of highly polymorphic among diverse germplasms provide some advantages in genetics and breeding applications. In spite of considerable efforts in developing molecular markers in maize, the number of SSRs publicly available is still limited. From >260 000 SSRs identified from 17 tested genomes, we detected 111 887 SSR loci with unique flanking sequence and single binding site through genome sequence blast and e-PCR analysis. These SSR loci can be developed as polymorphic markers *in silico* and public on the MaizeGDB database, which are ∼60 times more than those deposited in the MaizeGDB database so far. A total of 1556 SSR markers from the MaizeGDB database have specific location, and 16.6% of the newly developed SSR markers shared the same loci with public SSR markers. For some of the public SSR markers, the amplicon size was too large and it contained several newly developed SSR primers with different motifs. Therefore, only 0.47% (527) of newly developed SSR markers had completely compatible position with public SSR markers. Another reason for a few common SSRs shared with the two datasets maybe the traditional method for SSR marker development was based on screening of small-insert or microsatellite-enriched genomic libraries by hybridization in different materials,^32^ which was different from our analyses based on B73 reference genome and other resequenced genomes. Furthermore, the second-generation sequencing was different from the Sanger sequencing, which also lead to the differences. The experimental validation also proved to detect more alleles than the expected *in silicon* analysis due to diverse materials used in the study, but some SSR loci with little length differences were also hard to distinguish. The average marker density for the newly developed dataset reached one SSR per 14.7 kb in the B73 reference genome, indicating that maize is a highly polymorphic species.^33^ The availability of abundant SSR markers allows dramatic improvement in the efficiency of marker-assisted selection and fine mapping of QTL regions.

Previous studies have mainly focused on di-, tri-, and tetra-nucleotide SSRs, whereas mono-, penta-, and hexa-nucleotide SSRs have not drawn enough attention for marker development. We found that mono-nucleotide SSRs had much higher polymorphism rates than others, and penta- and hexa-nucleotide SSRs had relatively longer repeat units. Intron and UTR SSRs were more polymorphic than CDS SSRs due to low selective pressure in non-coding regions, which were consistent with previous reports.^22,34–36^ Experimental validation using 20 maize inbreds and 7 teosinte species showed that over 70% of the primer pairs could generate the target bands with length polymorphism, promising a great potential for the application of these SSR markers. In practice, it would be very powerful when they are used for genetic populations derived from various types of maize germplasm that were sampled for this study.

## Supplementary data

Supplementary data are available at www.dnaresearch.oxfordjournals.org.

## Funding

This work was supported by the National High Technology Research and Development Program of China (2012AA101104) to Y.L. and Y.X., Sichuan Youth Science and Technology Foundation of China (2012JQ0003), and the National Natural Science Foundation of China (31101162) to Y.L.

## Supplementary Material

Supplementary Data
