# Supplementary material for: Development and Characterization of Simple Sequence Repeat Markers Providing Genome-Wide Coverage and High Resolution in Maize
Source: DNA Res. 2013 Jun 26;20(5):497–509. doi: 10.1093/dnares/dst026 (PMC3789560; doi:10.1093/dnares/dst026)
Supplement: Supplementary Data [file supp_20_5_497__index.html]

Development and Characterization of Simple Sequence Repeat Markers Providing Genome-Wide Coverage and High Resolution in Maize — Supplementary Data 

# Development and Characterization of Simple Sequence Repeat Markers Providing Genome-Wide Coverage and High Resolution in Maize

## 

Supplementary Data

**Files in this Data Supplement:**

- Supplementary Data - Docx file
- Supplementary Table 1 - xls file
- Supplementary Table 2 - xls file
