# Supplementary material for: Development and Characterization of Simple Sequence Repeat Markers Providing Genome-Wide Coverage and High Resolution in Maize
Source: DNA Res. 2013 Jun 26;20(5):497–509. doi: 10.1093/dnares/dst026 (PMC3789560; doi:10.1093/dnares/dst026)
Supplement: Supplementary Data [file supp_dst026_dst026supp.docx]

**Supplementary data:** Table S1 List of developed SSR markers. Information provided in this supplemental table include chromosome, SSR motif, the number of meterials harboring the locus, forward and reverse primers, ePCR product locations as well as length and polymorphic information content (PIC) estimated in 17 maize genomes. Positions were based on maize B73 genome (www.maizesequence.org Release 4a.53).

Table S2 Primer sequences, physical positions, allele number and polymorphic information content (PIC) of the 151 randomly selected SSR markers. “-”indicates developed SSR markers failed to generate stable and clear bands. ^a^ expected PIC value *in silicon* analysis based on 17 tested genomes. ^b^ PIC value estimated in marker validation experiment using 27 tested lines. ^c^ PIC value estimated in marker validation experiment using 17 tested lines with genome sequencing information. Positions were based on maize B73 genome (www.maizesequence.org Release 4a.53).
